# Acquisition Parameters Influence Diffusion Metrics Effectiveness in Probing Prostate Tumor and Age-Related Microstructure

**DOI:** 10.3390/jpm13050860

**Published:** 2023-05-20

**Authors:** Alessandra Stella Caporale, Marco Nezzo, Maria Giovanna Di Trani, Alessandra Maiuro, Roberto Miano, Pierluigi Bove, Alessandro Mauriello, Guglielmo Manenti, Silvia Capuani

**Affiliations:** 1 Department of Neuroscience, Imaging and Clinical Sciences, ‘G. d’Annunzio’ University of Chieti-Pescara, 66100 Chieti, Italy; 2 Institute for Advanced Biomedical Technologies (ITAB), ‘G. d’Annunzio’ University of Chieti-Pescara, 66100 Chieti, Italy; 3 Interventional Radiology Unit, Department of Biomedicine and Prevention, Tor Vergata University of Rome, 00133 Rome, Italy; marco.nezzo@ptvonline.it; 4 Centro Fermi–Museo Storico della Fisica e Centro Studi e Ricerche Enrico Fermi, 00184 Rome, Italy; mgiovanna.ditrani@gmail.com; 5 CNR ISC, c/o Physics Department, Sapienza University of Rome, Piazzale Aldo Moro 5, 00185 Rome, Italy; alessandra.maiuro@uniroma1.it; 6 Physics Department, Sapienza University of Rome, Piazzale Aldo Moro 5, 00185 Rome, Italy; 7 Division of Urology, Department of Surgical Sciences, Tor Vergata University of Rome, 00133 Rome, Italy; mianor@virgilio.it (R.M.); pierluigi.bove@uniroma2.it (P.B.); 8 Anatomic Pathology, Department of Experimental Medicine, PTV Foundation, Tor Vergata University of Rome, 00133 Rome, Italy; alessandro.mauriello@uniroma2.it; 9 Department of Biomedicine and Prevention, UOC Radiology PTV Foundation, Tor Vergata University of Rome, 00133 Rome, Italy; guglielmo.manenti@uniroma2.it

**Keywords:** prostate, DTI, Gleason score, aging, b-value, diffusion length

## Abstract

This study aimed to investigate the Diffusion-Tensor-Imaging (DTI) potential in the detection of microstructural changes in prostate cancer (PCa) in relation to the diffusion weight (b-value) and the associated diffusion length l_D_. Thirty-two patients (age range = 50–87 years) with biopsy-proven PCa underwent Diffusion-Weighted-Imaging (DWI) at 3T, using single non-zero b-value or groups of b-values up to b = 2500 s/mm^2^. The DTI maps (mean-diffusivity, MD; fractional-anisotropy, FA; axial and radial diffusivity, D// and D┴), visual quality, and the association between DTI-metrics and Gleason Score (GS) and DTI-metrics and age were discussed in relation to diffusion compartments probed by water molecules at different b-values. DTI-metrics differentiated benign from PCa tissue (*p* ≤ 0.0005), with the best discriminative power versus GS at b-values ≥ 1500 s/mm^2^, and for b-values range 0–2000 s/mm^2^, when the l_D_ is comparable to the size of the epithelial compartment. The strongest linear correlations between MD, D//, D┴, and GS were found at b = 2000 s/mm^2^ and for the range 0–2000 s/mm^2^. A positive correlation between DTI parameters and age was found in benign tissue. In conclusion, the use of the b-value range 0–2000 s/mm^2^ and b-value = 2000 s/mm^2^ improves the contrast and discriminative power of DTI with respect to PCa. The sensitivity of DTI parameters to age-related microstructural changes is worth consideration.

## 1. Introduction

Prostate cancer (PCa) is the second most common form of male cancer worldwide [1]. The early detection of small latent carcinomas is responsible for the relatively low ratio of PCa mortality to incidence [1]. However, ultrasound (US)-guided biopsy, the standard diagnostic procedure, presents a high false negative rate (with a 25% probability of missing multi-focal cancer) and is linked to patient discomfort [2,3]. The uncertainty in establishing tumor aggressiveness leads to over-treatment, causing radical prostatectomy when avoidable, with high relapse rates [4]. PCa lesions present with various degrees of histological alterations, and are classified based on the Gleason system, a scale ranging from Gleason score (GS) = 6 to GS = 10, with increasing tumor aggressiveness and worsening of prognosis [5].

Recently, MRI has been reconsidered in the diagnosis of PCa; the Guidelines on Prostate Cancer include multi-parametric MRI (mpMRI) as a reliable tool to assess PCa risk and determine the necessity of re-biopsy after a negative US-guided biopsy [6,7]. MpMRI is also the recommended modality for the local staging of prostate cancer [8]. Tumor detection rates with mpMRI, depending on the volume and histological grading of the tumor, are high for clinically significant cancer with Gleason Score (GS) ≥ 7 (negative predictive value varying in 63–98% for cancer core lengths of at least 3 mm [9]). According to the PI-RADS^TM^ v2 (Prostate Imaging–Reporting and Data System, v2–2015 [10]), diffusion-weighted imaging (DWI) is the dominant sequence to detect PCa in the peripheral zone (PZ) of the prostate gland, while T2-weighted imaging (T2wI) is the most important sequence for tumor detection in the transition zone (TZ) [11].

DWI is based on the acquisition of signals from diffusing water molecules along a chosen direction, within a selected observation window or diffusion time ∆, given a diffusion sensitizing gradient strength g and duration δ. These experimental parameters are condensed in a factor called b-value [12], which modulates the sensitivity of diffusion imaging to the slower or faster random dynamics of water molecules in tissues. The advantage of using strong diffusion gradients (or high b-values) in the study of cancerous lesions is that the diffusion-weighted signal of the tissue surrounding the tumor is more attenuated with respect to cancerous tissue [13], usually characterized by slower water dynamics. This benefit, however, is tempered by a worsening in the signal-to-noise ratio (SNR) and the presence of imaging artifacts due to eddy currents, more prominent when high magnetic field gradients are employed [12]. 

Past works [14,15,16] showed that DWIs acquired at b-values higher than 1000 s/mm^2^ can facilitate the detection of suspicious PCa regions in prostatic tissue. However, to distinguish PCa lesion aggressiveness, the apparent diffusion coefficient (ADC) and/or mean diffusivity (MD) must be obtained [17,18,19,20]. In the last decade, there has been much effort in establishing the potentiality of Diffusion Tensor Imaging (DTI) parameters such as MD and fractional anisotropy (FA) in the discrimination between benign vs malignant and low vs high GS lesions. For instance, Nezzo et al. showed the potential ability of MD quantified at high b-values (higher than 800 s/mm^2^) in discriminating between low and high Gleason Grades [21]. According to the PI-RADS^TM^ v2, there is no optimal ‘high b-value’, but b-values of 1400–2000 s/mm^2^ or higher seem to be advantageous [10], if adequate SNR permits, in that poor SNR affects the accuracy of DTI metric estimates [19]. However, to date, the reasons that lie beneath the diagnostic potential of DTI parameters depending on the chosen b-values, i.e., on the chosen diffusion length versus the size of the epithelial compartment, have not yet been discussed in the context of PCa.

In this work, we aimed to compare the visual quality and the diagnostic efficacy of DTI parametric maps using a mono-exponential model, as a function of the b-values and b-value ranges, and we discussed the results in the framework of water diffusion. Therefore, we considered how physiological aging and pathological, cancerous microstructural alterations modulate the diffusion contrast, with the goal of clarifying why a specific b-value or b-value range can effectively enhance PCa lesions and improve PCa grading. Our results indicated that the contrast and discriminative power of DTI with respect to PCa is maximized with the use of the b-value range 0–2000 s/mm^2^ and b-value 2000 s/mm^2^, and that the sensitivity of DTI parameters to age-related microstructural changes is worth consideration.

## 2. Materials and Methods

### 2.1. Patient Recruitment

Thirty-six patients (mean ± standard deviation of age = 71.1 ± 9.1 years, age range: 50–87 years) with a US-guided biopsy-proven PCa were retrospectively enrolled in this study, providing informed consent. According to the ethics committee regulations of our country, the Institutional Review Board (IRB) approval was not required for this work, since it was a retrospective study, and it was conducted while ensuring the anonymity and confidentiality of the data. The cohort of 36 patients underwent MRI examination and biopsy under medical prescription regardless of this study. The examinations were conducted in agreement with the standard urogenital radiology clinical routine, following the European Society of Urogenital Radiology guidelines.

### 2.2. MRI Protocol

In compliance with the updated Guidelines on Prostate Cancer [6], mpMRI was carried out after a minimum of 2 months from the first biopsy. MRI was performed at 3T without an endorectal coil (PI-RADS^TM^ v2 [10]). The scanner (Intera Achieva, Philips Medical Systems, The Netherland, B.V., Eindhoven) was equipped with high-performance gradients (maximum strength = 80 mT/m, slew rate = 200 mT/m/ms), and with a six-channel phased array SENSE-TORSO radiofrequency (rf) coil. The patients were scanned head-first supine, with the rf coil placed on the pelvic area, at the pubic symphysis. T2-weighted (T2wIs) and diffusion-weighted axial images (DWIs) were obtained for each patient covering the entire prostate. A T2-weighted turbo spin echo sequence was used with repetition time/echo time, (TR/TE) = 3957/150 ms, turbo factor 21, field of view (FOV) = 150 × 130 mm^2^, slice thickness (STK) = 3 mm, no interslice gap, acquisition matrix = 256 × 178, reconstruction matrix = 512 × 512, number of signals averaged (NSA) = 6, flip angle (FA) = 90°. DWIs were acquired with single-shot Echo Planar Imaging (EPI; TR/TE = 3000/67 ms, ∆ = 50 ms, FOV = 150 × 130 mm^2^, STK = 3 mm, interslice gap = 0.3 mm, acquisition matrix = 64 × 52, reconstruction matrix = 96 × 96, NSA = 4), including 5 b-values (500, 1000, 1500, 2000, 2500 s/mm^2^) and 6 non-coplanar gradient diffusion directions, plus a volume acquired without diffusion weight (b = 0 s/mm^2^), named the b0-image. SPectral Attenuated Inversion Recovery (SPAIR) fat suppression with 200 Hz frequency offset was used after B_0_ homogeneity optimization, via high-order shimming routine. All images were anonymized and transferred to a workstation in DICOM format for subsequent analysis. 

The acquisition time for each patient was 12 min, with the DTI protocol lasting approximately 9 min. Two patients out of 36 were excluded due to consistent motion artifacts in the DWIs. Thus, the study group was reduced to 34 patients.

### 2.3. Image Analysis and DTI-Metrics Quantification

FSL 5.0 software (FMRIB Software Library v5.0, FMRIB, Oxford, UK [22]) was used for image processing. DWIs were co-registered to the b0-image through a rigid-body transformation with 6 degrees of freedom, correcting at the same time for motion artifacts and eddy current induced image distortions. DTIFIT was used to extract the diffusion tensor, modeling the signal attenuation as a mono-exponential decay. DTI parameters (mean diffusivity, MD, fractional anisotropy, FA, axial and radial diffusivities, respectively, D// and D┴) were first obtained using the b0 and each DWI, taking the b-values singularly (500, 1000, 1500, 2000, 2500 s/mm^2^), and then considering the b0 and four different sets of b-values: (0, 500, 1000) s/mm^2^, i.e., b-values range 0–1000 s/mm^2^; (0, 500, 1000, 1500) s/mm^2^, i.e., b-values range 0–1500 s/mm^2^; (0, 500, 1000, 1500, 2000) s/mm^2^, i.e., b-values range 0–2000 s/mm^2^; (0, 500, 1000, 1500, 2000, 2500) s/mm^2^, i.e., b-values range 0–2500 s/mm^2^.

By using MD it is possible to obtain an estimate of the diffusion length l_D_ traveled on average by water molecules during the observation window ∆, also named the root mean squared displacement (RMSD) [23,24]. The RMSD quantifies the intrinsic resolution of the DWI investigation at the various b-values (for details on the diffusion modeling used and RMSD see Appendix A). 

77 axial slices in total were used to evaluate DTI parameters in PCa areas, both in PZ and in the central gland (CG, comprising transitional zone and central zone), and the contra-lateral area with respect to cancer was taken as a reference region of non-cancerous, benign prostatic tissue. Regions of interest (ROIs) were manually drawn in PCa and in the ctrl area on the b0-image by two experienced uro-radiologists (M.N. and G.M.) blinded to the patient’s information, using the T2wIs as an anatomical reference. Inclusion criteria for ROIs were the absence of calcifications, hemorrhage, necrosis, and neurovascular bundles, and a minimum size of 4 pixels, corresponding to a lesion surface of at least 8 mm^2^. DTI parameters were evaluated in the ROIs with custom-made MATLAB scripts (MATLAB R2016a, The MathWorks Inc., Natick, MA, USA). The diffusion parameters were averaged over groups of age-matched patients for the analysis of the relationships between DTI metrics and age.

### 2.4. MR-Targeted Biopsy and Classification of PCa Lesions

We ensured that none of the patients with malign histopathological findings had been treated with either radiotherapy or hormone therapy before the biopsy. Indeed, these treatments may lead to an artificial elevation of the GS, due to the collapse of glandular architecture (see [25] and references within).

T2wIs were used as a reference image to delineate lesion boundaries using dedicated software (Watson Elementary^®^, Oncology Systems Limited 14 Longbow Close, Shrewsbury Shropshire, SY1 3GZ UK). Two to four cores were produced via targeted MR/ultrasound fusion biopsy (BiopSee ^®^, Medcom, Darmstadt, Germany); in the same session, performed by a different physician (unaware of the MR findings), 12 cores were produced via transperineal biopsy (sextant and laterally directed biopsies at base, mid-gland, and apex). Histopathological examination was performed and reviewed for each specimen based on the recommendations arising from the ‘consensus conference ISUP 2014′ [5]. The lesions were assigned GS ranging from GS = 3 + 3 to GS = 5 + 4 (see Table 1). Furthermore, the lesions were distinguished in 3 levels based on the low-intermediate-high classification by Hambrock et al. [26]: low-grade lesions (L), with Gleason grade 3 components only (in this study, GS = 3 + 3); intermediate-grade lesions (I), with a primary grade < 4 and a secondary grade 4 component (in this study, GS = 3 + 4); and high-grade lesions (H), with a primary grade 4 and a primary or secondary grade 5 component (in this study, GS = 4 + 3, GS = 4 + 4, GS = 4 + 5, GS = 5 + 4). Two patients with malignant focal lesions less than 5 mm in maximal diameter in the histopathologic specimen were excluded from the study group, which was finally comprised of 32 patients.

### 2.5. Signal-to-Noise Ratio, Contrast Ratio, and Coefficient of Variation

The signal-to-noise ratio (SNR) of DWIs at different b-values and diffusion directions was quantified as the ratio between the mean of the signal in a ROI (in the benign or control ctrl, and cancerous tissue, PCa) and the standard deviation (SD) of the signal from an artifact-free ROI placed in the rectum [27]. A subset of 5 patients for whom the rectum was clearly visible was considered for this purpose. The contrast-to-noise ratio (CNR) of DWIs was computed in the same subset of patients as

CNR_DWI_ = |I_PCa_ − I_ctrl_|/σ_bkg_, (1)

with I_PCa_ and I_ctrl_ being the signal intensities averaged on a lesion and the contralateral normal tissue, divided by the SD of the signal from the artifact-free ROI in the rectum [28]. In addition, we considered another common metric in prostate DWI studies at high b-value [14,15,16], the tumor-to-normal tissue contrast ratio (CR) in DWIs, computed as

CR_DWI_ = |I_PCa_ − I_ctrl_|/(I_PCa_ + I_ctrl_). (2)

The tumor to normal tissue CR of MD and FA maps was obtained using the relation

CR_(MD/FA)_ = |I_PCa_ − I_ctrl_|/sqrt(σ_PCa_^2^ + σ_ctrl_^2^), (3)

as reported in [29]. The histograms of CR_MD/FA_ were realized considering 10 monospaced bins. Finally, the percentage coefficient of the variation CV% of the DTI parameter estimates was computed as CV_%_ = SD/x_M_·100, where x_M_ is the mean value of a specific parameter in PCa, averaged over subjects with the same GS, with SD being its standard deviation.

### 2.6. Statistical Analysis

Non-parametric Friedman and Wilcoxon post-hoc tests with Bonferroni correction were used to compare the CR of DTI parametric maps. The association between the DTI parameters and GS was tested using Spearman rank correlation analysis and Pearson’s linear correlation analysis. The role of age in the DTI parameters variation was investigated by means of Pearson’s correlation test, assuming a linear correlation at first approximation. The multivariate analysis of variance (MANOVA) was performed with SPSS software (IBM SPSS Statistics for Windows, Version 20.0. Armonk, NY, USA: IBM Corp.), to check the discriminative effectiveness of DWI intensity and DTI parameters between benign and PCa tissue at different b-values, b-value ranges, and GS. For this purpose, DWI intensity in the cancer lesion was normalized to that of the contralateral tissue (control, ctrl), defined as I_N_ = (I_PCa_ − I_ctrl_)/I_ctrl_, to account for between-subject differences. 

In the investigation on the sensitivity of DTI parameters, tumor aggressiveness was considered once as a factor with 6 levels or GS, and then as a factor with 3 levels, based on the low-intermediate-high classification by Hambrock et al. [26].

An adjusted significance level P was considered to correct for multiple correlations and multiple comparisons. ROC curves were evaluated for the DTI parameters in the discrimination between PCa lesions and non-cancerous tissue, and between low and high tumor grades.

## 3. Results

### 3.1. Visual Quality of DWIs and DTI Parametric Maps of Prostate with Lesions

**Figure 1:**
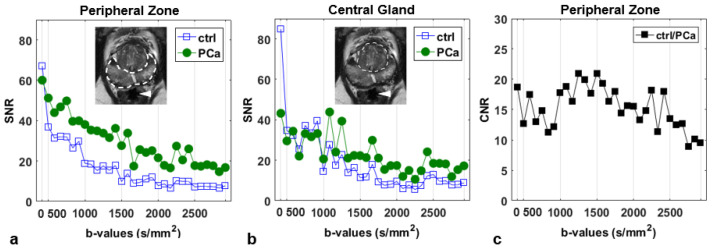
Signal-to-noise ratio (SNR) in the peripheral zone (**a**) and central gland (**b**) in contralateral non-cancerous tissue (ctrl) and cancerous tissue (PCa), and contrast-to-noise ratio (CNR) in the peripheral zone (PZ) (**c**). The peripheral and central zones are indicated by dashed lines in the inserts in (**a**,**b**), respectively. SNR was averaged on a subset of five patients with a free-artifacts rectum, which was used to estimate the noise (arrowhead). The b-values are placed on a discrete scale, varying between 0 and 2500 s/mm^2^, whereas the direction of the sensitizing diffusion gradient varies within two successive markers.

The SNR of DWIs is plotted as a function of b-value in Figure 1. SNR decreased with increasing diffusion weight, as expected, and it varied depending on the diffusion direction. For lesions in the PZ, SNR was approximately 60 in the b0-image, and it decreased up to 20, for b = 2500 s/mm^2^ (Figure 1a), whereas lesions in the CG presented a lower SNR (Figure 1b), still above the tolerance threshold for DWI up to the highest b-value [30]. We noticed that SNR was generally higher for PCa lesions compared to the benign tissue (control, ctrl).

The different contrast metrics evaluated on DWIs and DTI parameters are listed in Table 2. The highest CNR_DWI_ was achieved at b-values 1000 and 1500 s/mm^2^, in agreement with Metens et al. [28], and above 1000 s/mm^2^ CNR_DWI_ seemed to decrease progressively with the b-value. CR was quantified in DWIs and averaged over the six diffusion directions. There was no statistically significant difference between CR_DWI_(b = 2000) and CR_DWI_(b = 2500) (*p* > 0.005, *p* corrected for multiple comparisons); CR_DWI_(b = 2000) was significantly higher than CR(b = 1500), with *p* = 0.001, and CR_DWI_(b ≥ 1500) was significantly higher than CR_DWI_(b < 1500), with *p* < 0.0001. CR_MD_(b = 500) was significantly lower compared to all other b-values (*p* ≤ 0.0001, with *p* < 0.0005 for CR_MD_(b = 2000)). Different from DWIs, in MD maps the average CR was comparable for every b-value ≥ 1000 s/mm^2^, with the maximum reached for b = 2500 s/mm^2^. Considering the b-value ranges, CR_MD_(b = 0–2500) was significantly lower than all other b-ranges (*p* < 0.0001), and CR_MD_(b = 0–1500) was higher than CR_MD_(b = 0–2000) with *p* < 0.005. 

**Figure 2:**
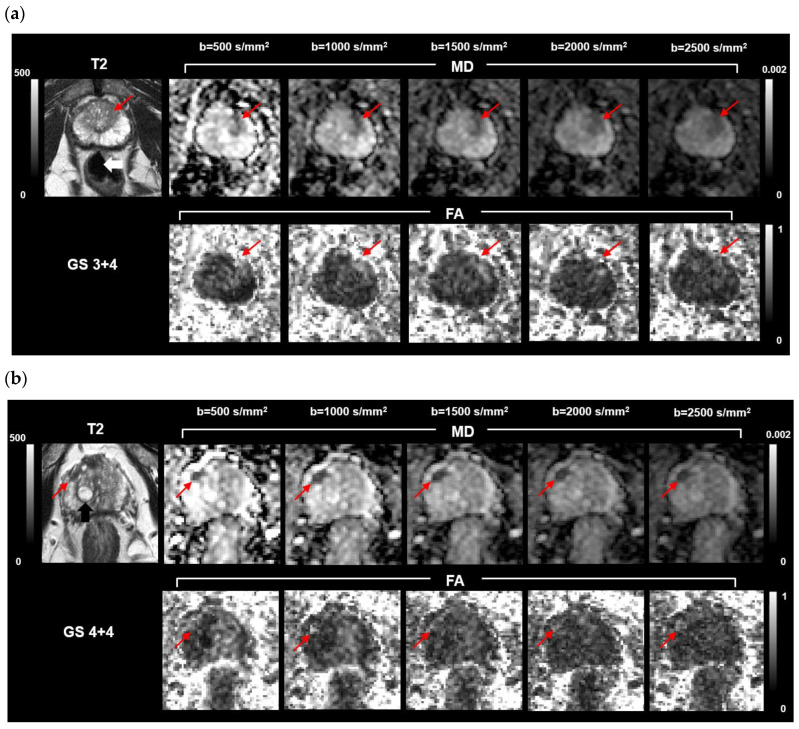
Parametric maps of two patients whose targeted biopsy revealed lesions in the left central gland with GS = 3 + 4 (**a**), and in the right peripheral zone with GS = 4 + 4 (**b**). Axial T2-weighted maps, MD, and FA maps are displayed for increasing b-values. The color bars indicate the intensity of T2-weighted maps in arbitrary units (left) and MD intensity expressed in mm^2^/s, and FA, unitless (right). The lesions (red arrows) appear hypointense in both the T2 and MD maps, and slightly hyperintense in the FA maps. A cyst full of liquid appears hyperintense in the T2 maps (black arrow); the rectum is also visible (white arrow).

Histograms of CR computed in MD and FA maps at different b-values and b-value ranges are reported in Figure S1 (Supplementary Materials). When visualized in MD maps at b = 2000 s/mm^2^, about 60% of lesions were detected with a CR_MD_ > 4.6, and 30% were detected with a CR_MD_ = 4.6. When visualized in MD maps derived using the b-value range 0–1500 s/mm^2^, about 80% of lesions were detected with CR_MD_ ≥ 4.1. On the other hand, PCa lesions were poorly contrasted towards non-tumoral tissue in FA maps, regardless of what b-value or b-range were used. These results can be more easily appreciated by considering the visual conspicuity of PCa lesions in MD and FA parametric maps from two patients, shown in Figure 2. PCa lesions were poorly contrasted towards healthy tissue in the grainy FA maps.

### 3.2. Discrimination of PCa Lesions Versus Non-Cancerous Tissue

**Figure 3:**
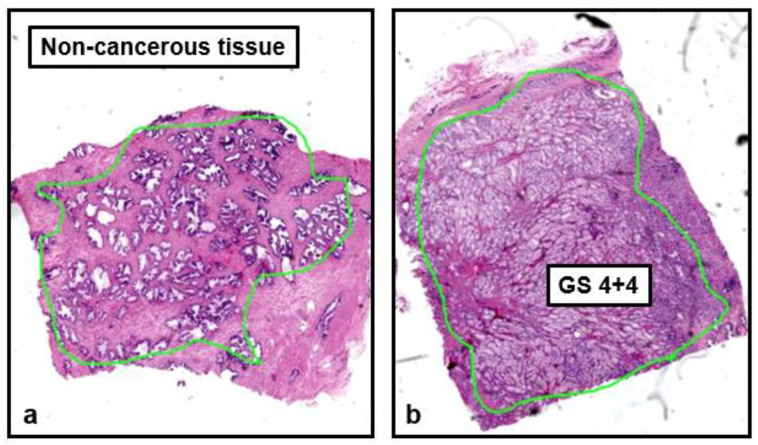
Prostate glandular structure in non-cancer tissue (**a**) versus cancer tissue, of Gleason Score 4 + 4 (**b**). The two specimens were obtained after prostatectomy. In (**a**) the glandular structure of the tissue is preserved (area delimited by the light green border); in contrast, the poorly formed or fused glands in (**b**) are typical of a lesion with Gleason Score 4 + 4.

Cancer-induced microscopic alterations in the structure of prostatic tissue can be appreciated in the histology reported in Figure 3, where non-cancerous tissue is compared to a lesion of GS = 4 + 4. 

**Figure 4:**
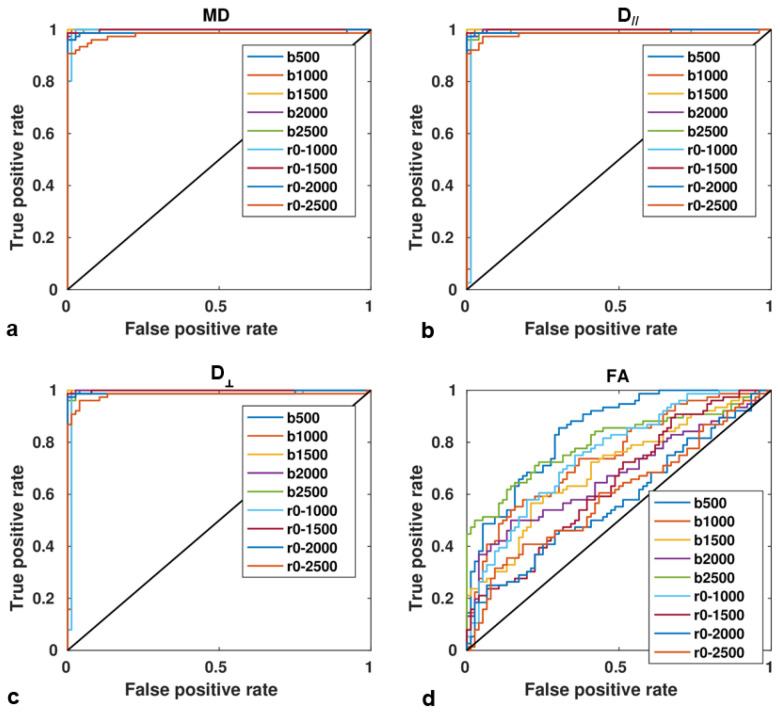
ROC curves showing the diagnostic ability of DTI metrics ((**a**), MD = mean diffusivity, (**b**), D//=axial diffusivity, (**c**), D┴ = radial diffusivity, (**d**), FA = fractional anisotropy) in the discrimination between PCa lesions and contralateral non-cancerous tissue, for each b-value (ranging from 500 to 2500 s/mm^2^) and b-value range (0–1000 to 0–2500 s/mm^2^).

Normalized intensity in DWIs (I_N_) discriminated well between PCa and benign tissue for all b-values (*p* < 0.0001). Similarly, DTI parameters discriminated between contralateral non-cancerous tissue and PCa, regardless of the b-value and b-value range. The ROC curves (Figure 4) showed that diffusivity metrics MD, D//, D┴ provided a very good discrimination between non-cancerous and PCa tissue for the entire set of b-values and b-value ranges considered, with accuracy, specificity, and sensitivity values close to 1 (Table S1, Supplementary Materials), and MD, D//, D┴ significantly lower in PCa lesions than control tissue, with *p* < 0.0001. The best accuracy was achieved at b-value = 1500 s/mm^2^ for diffusivity metrics (MD, D//, D┴) and at b-value = 2500 s/mm^2^ for FA. 

MD in the PZ healthy tissue was MD_PZ_ = (1.390 ± 0.180)·10^−3^ mm^2^/s) at b = 1500 s/mm^2^, and MD_PZ_ = (1.672 ± 0.245)·10^−3^ mm^2^/s at b = 1000 s/mm^2^, in agreement with the Literature [17,28]; instead, the cancerous lesions presented a reduced MD on average equal to MD_PCa_ = (0.677 ± 0.103)·10^−3^ mm^2^/s. The diffusion attenuated signal fitted to the mono-exponential model in the widest range of b-values (0–2500 s/mm^2^) provided the lowest value of MD in ctrl and PCa tissue: MD_PZ_ = (0.793 ± 0.137)·10^−3^ mm^2^/s and MD_PCa_ = (0.474 ± 0.056)·10^−3^ mm^2^/s (in agreement with Metens et al. [28]). The estimate of MD in PCa lesions showed a precision proportional to the b-value (i.e., the coefficient of variation CV% decreased, in parallel to the b-value increase; see Figure S2 in Supplementary Materials).

Considering both PZ and CG and averaging over all b-values, we measured FA_ctrl_ = 0.222 ± 0.061, with FA_PZ_ = 0.220 ± 0.066 and FA_CG_ = 0.229 ± 0.053 in non-cancerous tissue. Despite the poor contrast of FA maps, FA was significantly higher in PCa lesions vs ctrl, with *p* < 0.0001 for all b-values, *p* < 0.0001 for 0–1000 s/mm^2^, *p* < 0.0005 for 0–1500 s/mm^2^, and *p* < 0.005 for 0–2000 s/mm^2^ and 0–2500 s/mm^2^.

### 3.3. Discrimination between Different Degrees of Malignancy

The normalized intensity I_N_ of DWIs was not able to discriminate among GS for any b-value (*p* > 0.05). Instead, DTI parameters were effective in distinguishing between low/intermediate-grade lesions (GS = 3 + 3/3 + 4) and high-grade lesions (GS = 4 + 4/4 + 5/5 + 4), as reported in Table 3. The discriminative effectiveness of DTI parameters was dependent on the b-value, being stronger for the single b-value ≥ 1500 s/mm^2^. None of the considered b-values, nor ranges, ensured that DTI parameters could distinguish all the consecutive couples of GS in the studied cohort of patients.

The best accuracy for the discrimination between low- and high-grade cancer, represented by the highest area under the ROC curve (AUC), was achieved at b-value = 2000 s/mm^2^ for DTI metrics (Table 4). Except for FA, that generally showed scarce accuracy, slightly better performances were obtained when b-value range = 0–2000 s/mm^2^ was considered (Figure S3, Supplementary Materials).

### 3.4. Correlation between DTI Parameters and Gleason Score (GS)

**Figure 5:**
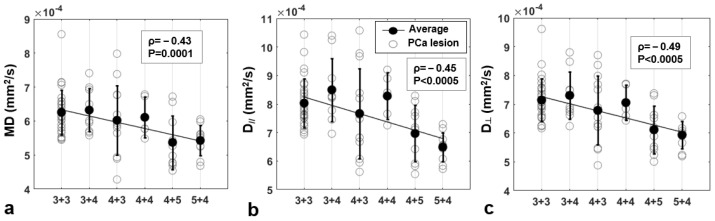
Correlation between DTI-metrics and GS at b = 2000 s/mm^2^. Mean Diffusivity (MD, (**a**)) axial diffusivity (D//, (**b**)) and radial diffusivity (D┴, (**c**)) are quantified in PCa lesions for each patient (experimental points, empty circles), and plotted as a function of the Gleason Score. The diffusion parameters averaged over each sub-group are drawn as solid circles. Spearman’s correlation coefficients are indicated in the boxes; regression line is plotted in black.

The values of Spearman’s significant correlations between DTI parameters and GS are moderate-low (see Table 5; Pearson’s correlations are reported in Table S2, Supplementary Materials). MD, D//, and D┴ derived using a single b-value showed the strongest significant correlations for b = 2000 s/mm^2^ (adjusted *p* = 0.0014). A significant negative correlation between MD and tumor GS was found for b ≥ 1500 s/mm^2^; D// and D┴ were negatively related to GS with *p* < 0.0005 for b ≥ 2000 s/mm^2^ (Figure 5). No significant linear correlations were found between FA and GS. MD, D//, and D┴ showed statistically significant correlations with GS for every b-value range except for 0–1000 s/mm^2^.

### 3.5. Association between the DTI Parameters and Patients’ Age

**Figure 6:**
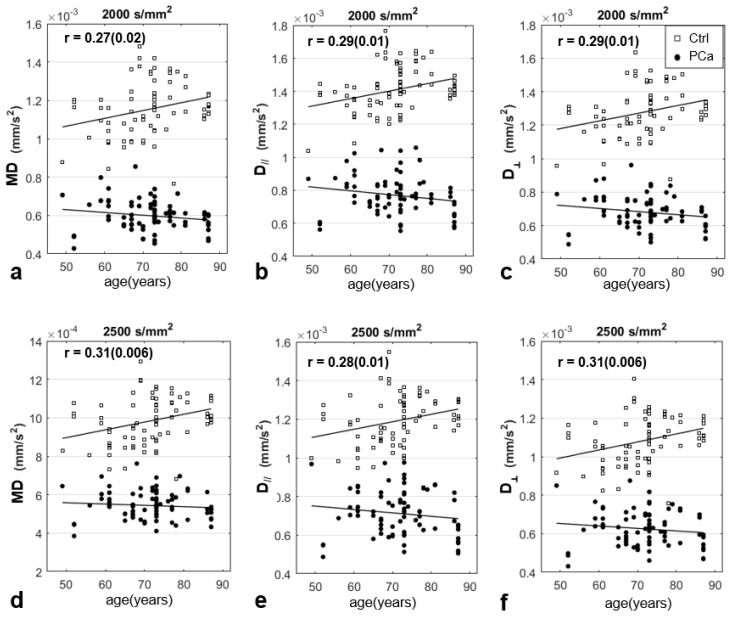
DTI parameters and prostate aging. Mean diffusivity (MD, (**a**,**d**)), axial (D//, (**b**,**e**)), and radial diffusivity (D┴, (**c**,**f**)) evaluated using distinct b-values, in each PCa lesion and contralateral non-cancerous ROI (control, ctrl), plotted as a function of patients’ age, for two distinct b-values (b = 2000 and b = 2500 s/mm^2^); only the significant correlations are shown. Pearson’s correlation coefficients (r) are indicated (P is in parenthesis). Regression line is plotted in black.

In Figure 6, MD, D//, and D┴ evaluated in PCa lesions and in contralateral non-cancerous tissue are displayed, as a function of the patients’ age, for the cases where significant associations between DTI parameters and age were found. The correlations between DTI-metrics and age were significant for the non-cancerous tissue only, considering the distinct b-values. FA did not show significant correlations with age for any considered b-value or b-value range. The GS of the lesions showed a non-significant, positive trend with the age of the patients, considering the 77 lesions (r = 0.22, *p* = 0.06).

## 4. Discussion

We investigated how the selection of diffusion weights or b-values in diffusion-weighted imaging (DWI) of the prostate affects the diagnostic potential of the derived parameters, concerning PCa enucleation against non-cancerous tissue and the discrimination between differently graded tumors. 

In DWI studies it is critical to ascertain that the SNR of the images is sufficiently high. In fact, the estimate of DTI-related parameters, particularly FA, is considerably affected by poor SNR [19,31,32]. Simulations on brain DWIs suggested DW-data to be reliable for SNR above 10 [33]. Figure 1 ensures that our results are not affected by noise bias and suggests that the SNR in PCa is generally higher than in the non-cancerous tissue, probably due to the reduced diffusivity in PCa lesions. In CG, which is more heterogeneous compared to PZ [34], the SNR was slightly lower than in PZ lesions but remained above the tolerance threshold for the SNR in DWI [30] up to the highest b-value (Figure 1b).

In terms of the contrast between cancerous and non-cancerous tissue, the DWIs acquired at b-values 1000 and 1500 s/mm^2^ showed the highest CNR, in agreement with Metens et al. [28], with CNR roughly doubled in our case, probably due to noise evaluated in a different region (the pelvis bone). The CNRs we obtained are similar to those measured by Kitajima et al. [35]. Moreover, we found similar CR for b = 2000 and b = 2500 s/mm^2^, which were both higher than those obtained at lower b-values. The increase in CR_DWI_ with the b-value may be explained by considering that the signal from the non-cancerous tissue is increasingly attenuated at higher b-values. However, increasing the b-value indefinitely does not necessarily translate into improved detection, as shown by some authors [14,15,16]; by artificially computing high b-values DWIs based on a mono-exponential model [36], the simulations showed that the tumor delineation was clearer at b-value 2000 s/mm^2^ compared to higher b-values, with similar tumor detection rates. Our results agree with Vural et al. and Rosenkranz et al. [15,16], and indicate that some care should be taken in measuring and comparing the contrast between cancerous and healthy tissue in DWIs. The PCa lesions appeared hypointense in MD maps with respect to the surrounding non-cancerous areas, as expected [17]. The average CR of MD maps seemed to be comparable for every b-value ≥ 1000 s/mm^2^, with the maximum at b = 2500 s/mm^2^; regarding the b-value ranges, CR_MD_ (b = 0–1000) was significantly higher (*p* < 0.0001) than CR_MD_ (b = 0–2500). We conjecture that the decrease in CR in DTI-maps obtained using b-value ranges 0–2000 and 0–2500 s/mm^2^ compared to narrower b-value ranges was due to the mono-exponential diffusion model adopted here. Indeed, it is well known that in biological tissues, the signal decay associated with water diffusion deviates from the mono-exponential behavior at b-values higher than approximately 1500 s/mm^2^, and it would be consequently better described by non-Gaussian diffusion models, such as the Kurtosis [37,38,39].

The coefficient of variation (CV%) relative to MD and D┴ in groups of lesions with known GS (Figure S2) decreased, indicating greater precision, with increasing b-values. This can be explained by considering that at low b-values the technique probes the diffusivity of bulk water, which is itself a source of high variability. At higher b-values, instead, the sensitivity of the technique is boosted towards slower diffusive motions, associated with the membrane-interacting water molecules [40], for example, those confined inside the 15 µm-thick epithelial compartments of glandular acini and ducts [41]. 

The cancerous lesions showed significantly reduced MD compared to non-cancerous tissue (*p* < 0.0001). The values obtained in PZ lesions are in agreement with the Literature (specifically, at b = 1500 s/mm^2^, MD_PZ_ = (1.390 ± 0.180)·10^−3^ mm^2^/s in agreement with Metens et al. [28], and MD_PZ_ = (1.672 ± 0.245)·10^−3^ mm^2^/s at b = 1000 s/mm^2^, in agreement with Gurses et al. [17]).

Considering the b-value range 0–2500 s/mm^2^, MD in PCa lesions was on average MD = 0.474 ± 0.056∙10^−3^ mm^2^/s, which is 40% lower compared to the contralateral part (MD = (0.793 ± 0.137)·10^−3^ mm^2^/s). The diminished water mobility in cancer tissue had been attributed to increased cellularity in the tumor, due to both cell swelling and cancer cell proliferation [42]. Based on the behavior of water molecules exploring diffusive compartments, we suggest a supplementary interpretation. The prostate glandular tissue presents three principal compartments, with progressively decreasing diffusivity: the ductal/acinal lumen, the stroma, and the epithelium. In vitro experiments on fixed normal prostate tissue showed that the variations in the relative amounts of tissues explain more than 50% of the variation in diffusivity [43]. Furthermore, the adenocarcinoma with Gleason patterns (Gp) three, four, and five is characterized by an increase of up to 30% in epithelium volume compared to the benign tissue, at the expense of the high diffusivity compartments (the stroma and the lumen, for which the volume is reduced by about 13–15% each) [42]. Therefore, MD decrease in PCa might be linked to the new arrangement of volumetric fractions among diffusive compartments, i.e., the 30% volumetric increase in the low-diffusivity epithelial compartment on one side, and the reduction of about 10% of the stromatic and glandular components.

**Figure 7:**
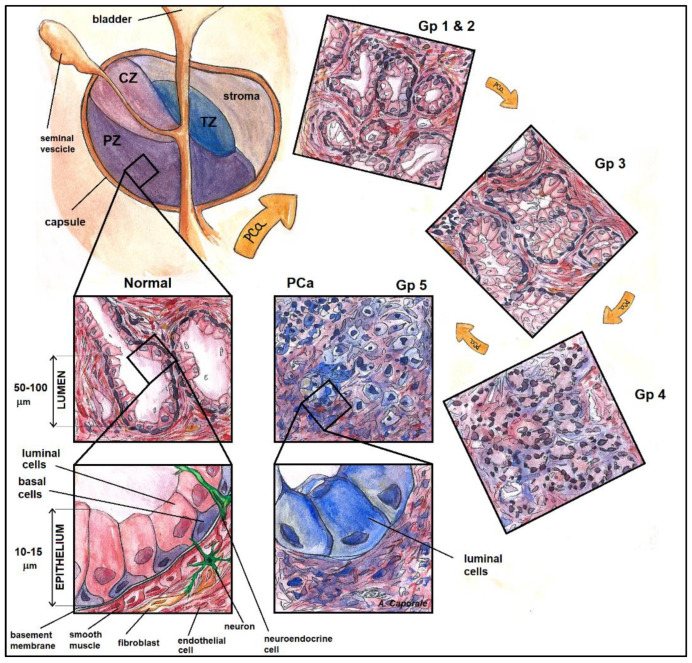
Illustration of the structural modifications brought about by the cancerogenic process. The sketch highlights the size of the various compartments that the water can explore during its random motion. Illustration realized by A. Caporale. PZ = peripheral zone; CZ = central zone; TZ = transition zone; PCa = prostate adenocarcinoma; Gp = Gleason pattern.

PCa lesions were well discriminated against non-cancerous tissue by MD at every considered b-value and b-values range (*p* < 0.0001). The range of diffusion lengths sampled in our study spanned from l_D_~10 µm at b = 2500 s/mm^2^ to a maximum of l_D_~25 µm (at b = 500 s/mm^2^). Water molecules traveling such distances are sensitive to the architectural rearrangement of the prostate due to carcinogenesis. Indeed, PCa is characterized by a gradual reduction in the glandular lumen that starts at the first stages of tumor development (Gp three), with a total loss and no glandular differentiation at its final stages (Gp five, Figure 7) [44]. While in the healthy prostate, the size of acini is at least four times l_D_, in PCa lesions it is drastically reduced because of the fusion of the glands or no gland formation. In fact, the luminal space is invaded by abnormally proliferating epithelial cells belonging to the luminal cell layer, at the expense of the basal cells layer, which gradually disappears [45].

DTI-metrics (MD, D//, and D┴) derived using a single b-value reflected the microstructure of the cancerous tissue for b ≥ 1500 s/mm^2^, and showed the most significant correlation with GS for b = 2000 s/mm^2^. There was a significant monotonic decrease in MD, D//, and D┴ with GS at every range of b-values (see Table 5 and Table S2), in agreement with Nezzo et al. [21]. However, MD, D//, and D┴ showed a significant monotonic decrease with GS in the ranges 0–1000 s/mm^2^ and 0–1500 s/mm^2^, but not for the single b-values b = 1000 s/mm^2^ and b = 1500 s/mm^2^, probably because in the first case a mono-exponential fit over a range of b-values (up to b = 1500 s/mm^2^) was performed, which is equivalent to consider a spectrum of diffusive motions, from the bulk water contribution to the slow diffusing water pool, in the epithelial compartment. No significant linear correlations were found between FA and GS, in agreement with Uribe et al.’s simulations [19] but in disagreement with Li et al. [18]. In the latter, 32 different diffusion directions were used, resulting in increased sensitivity of diffusion anisotropy towards the microstructure of cancerous tissue. In fact, FA is particularly sensitive to the number of diffusion directions used in the DTI protocols [46]. In addition, FA was found to be highly dependent on diffusion gradient separation, or Δ. Lemberskiy et al. found that FA increased in parallel with Δ, while MD and the apparent diffusion coefficient showed the opposite behavior [47]. These time dependencies were also observed in ex vivo prostate imaging at 9.4 T by Bourne et al. [48]. In our study, however, we kept Δ fixed and varied the b-value by increasing the diffusion gradient strength *g*.

ROC curve analysis conducted on the discrimination between low- vs. high-Gleason grades (Table 4, Figure S3) was in accordance with results obtained by the correlation analysis, where MD, D//, and D┴ showed the highest correlation with tumor aggressiveness when b-value range = 0–2000 s/mm^2^ was employed. 

The results of statistical tests comparing DTI parameters at different diffusion weights among GS ranging from 3 + 3 to 5 + 4, and between low, intermediate, and high grades of tumor are listed in Table 3. D// and D┴ often discriminated different GS that MD did not differentiate. Another study by Shenahr et al. [49] showed that the first diffusion eigenvalue discriminated well normal tissue from PCa, both in PZ and CG, with a higher mean difference between the tissues compared to ADC. Therefore, employing a DTI protocol, instead of simply estimating the mean ADC obtained along three orthogonal directions (x, y, z), yields a set of diffusion parameters that can discriminate more effectively PCa at different GS. At b = 2000 s/mm^2^, there was the highest number of significant contrasts for DTI parameters (except for FA). This is in agreement with Katahira et al., who compared the diagnostic performance of DWI at b = 2000 s/mm^2^ and b = 1000 s/mm^2^ in a group of 201 patients; the acquisition at high b-value provided a better sensitivity and specificity towards PCa lesion detection [13]. Considering both the number of contrasts and the level of significance, the ranges 0–1500 s/mm^2^, 0–2000 s/mm^2^, and 0–2500 s/mm^2^, combined with the mono-exponential model used in this study performed similarly.

These results may be explained considering that the distance traveled by water molecules at certain b-values is sensitive to l_D_~10 µm, a length comparable to the width of the epithelial compartment [41]. This may motivate the higher discriminative potential towards low- vs high-grade tumor of DTI parameters derived using b = 2000 s/mm^2^ or b-values range 0–2000 s/mm^2^. Indeed, the onset of the adenocarcinoma leads to structural modifications at the multi-cellular and sub-cellular level, which progressively alter the appearance of prostatic glandular and stromatic tissues, compared to the healthy prostate, as tumor aggressiveness increases (Figure 7). At the lowest clinically significant level (GS = 3 + 3), the alteration with respect to the benign tissue is moderate, with rare poorly formed glands, and a slight increase (less than 20%) in epithelium fractional volume [42]. Considering the diffusive length, it is reasonable to hypothesize that water molecules explore the acinar lumen in a regime of relatively free diffusion. In the intermediate grade lesions (GS = 3 + 4), the number of poorly formed glands increases, and there is a lesser component of cribriform/fused glands [50], but the predominant Gp = 3 likely causes this arrangement to be similar to the GS = 3 + 3 case, at least from the point of view of diffusing water molecules. It is in the lesions with a primary Gp of four or five (GS = 4 + 3/4 + 4/4 + 5/5 + 4) that the component of well-formed glands considerably drops, and the stromatic and lumen fractional volumes decreases by up to 15% each [42]. Due to the progressive shrinkage of the free diffusion compartment, water diffusion appears increasingly hindered and obstructed. 

Finally, the association between some DTI metrics and age indicated that some care should be taken when PCa diagnosis is performed with DTI. In fact, due to the dependence of diffusion parameters on age in a normally appearing prostate, the discrimination between benign and PCa tissue for younger patients might be less accurate (Figure 6). These results are in line with Zhang et al., where a significant positive relationship was found between ADC and age in healthy normal volunteers [51]. The relation between physiological aging and water diffusion in prostate tissue is ascribable to changes in stromal-epithelial interactions, alterations in the basal membrane composition, and eventually the occurrence of inflammatory processes.

**Figure 8:**
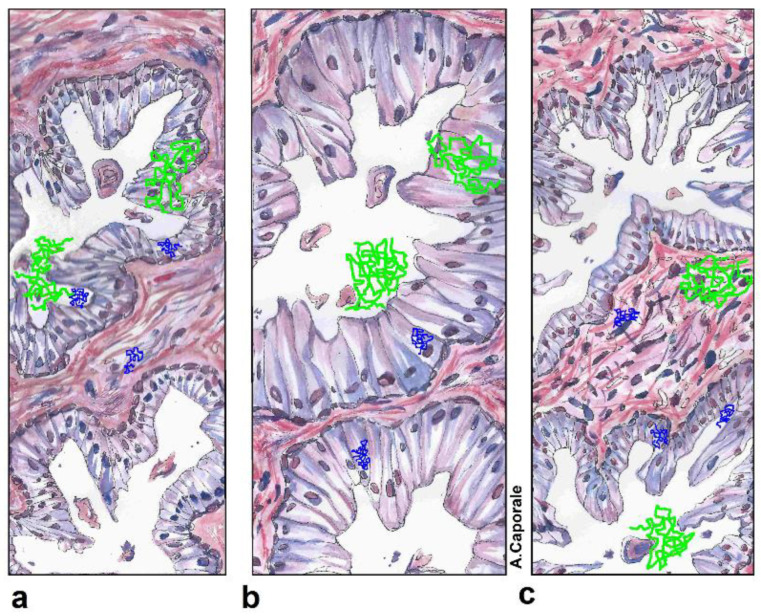
Fast and slow water diffusion dynamics and tissue structure in benign prostate. The drawing shows several possible random trajectories of water molecules superimposed on the histological appearance of prostate glandular tissue in three different cases: normal acinar tissue (**a**), hypertrophy of epithelial cells (**b**), and hyperplasia of epithelial and stromal cells (**c**). Only two acini are depicted for the sake of simplicity and in every case (different from the adenocarcinoma case, not shown) both the epithelial and the basal cells layers are preserved. The trajectories depicted in acid green are representative of the fast water dynamics to which the diffusion-weighted MRI (DW-MRI) is sensitive at b-value b = 500 s/mm^2^; the trajectories depicted in blue are representative of the slow water dynamics which may be assessed at high b-values (b = 2500 s/mm^2^). In the latter case, the DW-MRI provides finer structural information, at a length scale comparable to the size of the epithelial layer.

Apart from PCa, another condition altering the structure of the prostate gland is prostatic enlargement, a benign condition affecting over 90 million men globally, where the prostate is influenced by both hypertrophy and hyperplasia [52]. Benign prostatic hyperplasia (BPH) affects the CG and is usually accompanied by epithelial and stromal hyperplasia [53]. The term hypertrophy refers to the increment in cell volume or cell enlargement, whilst the term hyperplasia indicates the increase in cell number or cell proliferation. Therefore, due to hypertrophy, water mobility in the epithelial compartment will increase because of cell enlargement (Figure 8). On the other hand, it has been shown that the development of adenomatous nodules in prostatic hyperplasia is characterized by a significantly reduced epithelium-to-stroma ratio compared to normal prostate tissue, causing a shrinkage of the low diffusivity compartment in favor of the high diffusivity one. We suggest that these two factors could interplay in the physiological increase in water MD, D//, and D┴ due to age in benign prostatic tissue. 

Our study presents some limitations. First of all, we used histology based on biopsy as a reference technique to classify PCa lesions. Due to the known limits of the biopsy procedure we cannot ensure the perfect correspondence between the diffusion parameters extracted from a specific portion of the prostate and the GS derived from the respective biopsy.

Secondly, some considerations regarding the acquisition protocol should be made. The optimization strategy to minimize noise bias of MD and FA parameters and maximize the accuracy in the estimation of the diffusion tensor is still a matter of debate. The variables that play a major role are the number of b-values, the number and type of diffusion directions, and the number of repetitions for each acquisition. If in the case of the human brain, characterized by highly anisotropic areas (the white matter fibers), using optimized gradient schemes with more than six diffusion directions is appealing because it increases variance uniformity in the calculated DTI parameters and minimizes the effect of noise-bias, in clinical applications the use of more than six diffusion directions does not significantly affect image quality for the purpose of PCa diagnosis [46]. Indeed, fiber tractography in prostate parenchyma is feasible with just six diffusion directions [54], although in the Literature there are contrasting results regarding increased or decreased fiber tract density in the tumor with respect to the non-cancerous tissue [54,55]. For what concerns the b-values, it was suggested that increasing the number of repetitions at the optimum b-value is more useful than acquiring only once at different b-values, providing the most precise and least biased estimation of scalar indices derived from the diffusion tensor [56]; others showed that the use of multiple b-values improves diffusion parameter accuracy, as long as the minimum number of directions required for robust estimation of each parameter is used [57]. In our study performed at 3 T with six diffusion directions, we found that the discriminative power of DTI parameters as well as the correlation between diffusion parameters and GS was the highest for the single b-value b = 2000 s/mm^2^ or the range 0–2000 s/mm^2^. However, we did not evaluate the eventual improvement in the diagnostic performance of DTI parameters by repeating the acquisition multiple times at the optimal b-value.

Finally, since we probe the investigated tissue with the characteristic diffusion lengths, we conjecture that the use of single b-values higher than 2500 s/mm^2^ would not significantly improve the discriminative power of DTI parameters with respect to GS. In fact, at diffusion lengths l_D_ < 10 µm, the epithelial compartments where water diffusion is restricted may appear homogeneous among different Gleason patterns, thus reducing the variability of the extracted diffusion parameters. Another substantial difference between normal and neoplastic cells is the presence of prominent nucleoli (with a diameter > 1 µm) in the latter [25]. However, to make water displacements sensitive to such a difference, it would be necessary to increase considerably the b-value (up to b = 5000 s/mm^2^), adopting non-Gaussian diffusion models [39,58] or anomalous diffusion models [59,60,61,62,63]. Other strategies to improve the discriminative power of diffusion parameters consider the exploitation of both the diffusion and relaxation properties of tissue compartments, such as in a recently developed biophysical model, called rVERDICT, which allows to obtain MRI parameters with enhanced sensitivity and specificity with respect to the cancer-related tissue modifications [64].

## 5. Conclusions

The aim of this work was to discuss the ability of DTI in detecting PCa-related microstructural changes in dependence on the diffusion-weight or b-value. MD, D//, and D┴ and FA discriminated well PCa from benign tissue, for every b-value up to b = 2500 s/mm^2^; we suggest that this is linked to the changes in the relative size of diffusion compartments in cancer, for example, the abnormal development of the epithelium at the expenses of stroma and the lumen. When only six diffusion directions are used for the estimation of the diffusion tensor, the use of b = 2000 s/mm^2^ provides the best contrast in MD maps, while in FA maps the contrast remains poor. Regarding the discrimination between different degrees of malignancy, at b = 2000 s/mm^2^, MD, D//, and D┴ are effective in distinguishing low- from high-grade tumors and intermediate- from high-grade tumors, probably because the associated l_D_ are optimal to probe the tissue at scale lengths typical of epithelial compartments associated with cancer-related degeneration. Using 0–1500 and 0–2000 s/mm^2^ b-value ranges combined with a simple mono-exponential model seems ideal in prostate diffusion imaging. Furthermore, wider ranges of b-values would benefit from adopting a more sophisticated diffusion model, such as the kurtosis model. Finally, patients’ age does not affect DTI parameters in PCa, whereas it could play a role in benign tissue. This behavior may be a consequence of physiological hypertrophy and hyperplasia occurring in the aging prostate, which might increase the relative volume characterized by fast diffusion. 

## Figures and Tables

**Table 1:** PCa lesions evaluated in the study, their classification, and their location.

GS ^a^	Three Levels Grade (L, I, H) ^b^	Number of Patients	Number of PZ ^c^ Lesions	Number of CG ^d^ Lesions	Number of PZ + CG Slices
3 + 3	L	16	10 (17 slices)	6 (12 slices)	29
3 + 4	I	5	8 (7 slices)	5 (3 slices)	10
4 + 3	H	4	8 (13 slices)	0	13
4 + 4	H	3	5 (5 slices)	0	5
4 + 5	H	2	2 (10 slices)	0	10
5 + 4	H	2	1 (8 slices)	1 (2 slices)	10
Tot.		32	34 (60 slices)	12 (17 slices)	77

^a^ Gleason Score, GS; ^b^ L = Low, I = Intermediate, H = High grade; ^c^ Peripheral Zone, PZ; ^d^ Central Gland, CG.

**Table 2:** Contrast between PCa and contralateral non-cancerous tissue.

b-Value (s/mm^2^)	CNR_DWI_^a^	CR_DWI_	CR_MD_	CR_FA_	CR_MD_ b-Range ^b^	CR_FA_ b-Range ^b^
500	11.6 ± 2.0	0.13 ± 0.08	3.82 ± 0.18	1.03 ± 0.09	-	-
1000	15.4 ± 1.5	0.26 ± 0.12	4.48 ± 0.21	0.78 ± 0.07	4.32 ± 0.17	0.85 ± 0.08
1500	14.4 ± 2.0	0.33 ± 0.14	4.57 ± 0.17	0.68 ± 0.06	4.17 ± 0.17	0.79 ± 0.08
2000	12.7 ± 2.3	0.35 ± 0.15	4.44 ± 0.16	0.58 ± 0.07	3.79 ± 0.18	0.66 ± 0.07
2500	9.6 ± 1.6	0.34 ± 0.14	4.62 ± 0.20	0.74 ± 0.07	3.21 ± 0.18	0.60 ± 0.06

CNR = contrast-to-noise ratio; CR = contrast ratio; ^a^ Mean value ± standard deviation (SD) in the selected region of interest; ^b^ CR of MD and FA maps obtained with a mono-exponential fit over a set of b-values ranging from 0 to the indicated b-value.

**Table 3:** Discriminations between different Gleason Scores (GS) of PCa lesions by diffusion metrics evaluated at various b-values and b-value ranges. The significance level P is indicated in parenthesis.

b-Value (s/mm^2^)		M	D//	D┴	FA
500	GS	-	-	-	3 + 4/5 + 4 (0.03) 4 + 5/5 + 4 (0.02)
	LIH ^a^	-	-	-	-
1000	GS	-	-	-	-
	LIH	-	-	L/H (0.04)	-
1500	GS	-	3 + 4/5 + 4 (0.009)	3 + 4/5 + 4 (0.03)	4 + 5/5 + 4 (0.02)
	LIH	-	I/H (0.02)	L/H (0.04) I/H (0.02)	-
2000	GS	3 + 3/4 + 5 (0.02) 3 + 3/5 + 4 (0.04)	3 + 3/5 + 4 (0.002) 3 + 4/4 + 5 (0.02) 3 + 4/5 + 4 (0.0007) 4 + 4/5 + 4 (0.03)	3 + 3/4 + 5 (0.02) 3 + 3/5 + 4 (0.002) 3 + 4/4 + 5 (0.03) 3 + 4/5 + 4 (0.006)	3 + 3/5 + 4 (0.02)
	LIH	L/H (0.01)	L/H (0.01) I/H (0.003)	L/H (0.002) I/H (0.01)	-
2500	GS	3 + 3/5 + 4 (0.04)	3 + 3/5 + 4 (0.004) 3 + 4/5 + 4 (0.01)	3 + 3/5 + 4 (0.005)	3 + 4/5 + 4 (0.02)
	LIH	L/H (0.01)	L/H (0.009) I/H (0.03)	L/H (0.003)	-
b-values range (s/mm^2^)		MD	D//	D┴	FA
0–1000	GS	-	-	-	3 + 4/5 + 4 (0.007) 4 + 5/5 + 4 (0.045)
	LIH ^a^	-	L/H (0.04)	L/H (0.03)	I/H (0.02)
0–1500	GS	-	3 + 3/5 + 4 (0.007) 3 + 4/5 + 4 (0.003)	3 + 3/5 + 4 (0.006) 3 + 4/5 + 4 (0.004)	-
	LIH	L/H (0.03) I/H (0.03)	L/H (0.02) I/H (0.009)	L/H (0.003) I/H (0.003)	-
0–2000	GS	3 + 3/5 + 4 (0.02) 3 + 4/5 + 4 (0.045)	3 + 3/5 + 4 (0.001) 3 + 4/5 + 4 (0.0006)	3 + 3/5 + 4 (0.001) 3 + 4/5 + 4 (0.002)	3 + 4/5 + 4 (0.049)
	LIH	L/H (0.007) I/H (0.04)	L/H (0.02) I/H (0.006)	L/H (0.002) I/H (0.006)	-
0–2500	GS	3 + 3/5 + 4 (0.01)	3 + 3/5 + 4 (0.0006) 3 + 4/5 + 4 (0.002)	3 + 3/5 + 4 (0.001) 3 + 4/5 + 4 (0.02)	3 + 4/5 + 4 (0.006)
	LIH	L/H (0.008)	L/H (0.007) I/H (0.03)	L/H (0.002)	I/H (0.03)

^a^ L = Low, I = Intermediate, H = High grade.

**Table 4:** Area under the curve (AUC), specificity (Sp), and sensibility (Se) obtained from ROC curve analysis for the classification of low- and high-risk PCa.

**b-value (s/mm^2^)**	**MD**			**D//**			**D┴**			**FA**		
	**AUC**	**Sp**	**Se**	**AUC**	**Sp**	**Se**	**AUC**	**Sp**	**Se**	**AUC**	**Sp**	**Se**
500	0.59	0.9	0.37	0.66	0.84	0.47	0.65	0.89	0.45	0.61	0.42	0.79
1000	0.64	0.9	0.4	0.69	0.89	0.53	0.69	0.87	0.47	0.67	0.79	0.53
1500	0.68	0.97	0.42	0.71	0.87	0.53	0.72	0.82	0.63	0.61	0.76	0.53
2000	0.72	0.66	0.74	0.74	1	0.47	0.75	0.76	0.71	0.67	0.74	0.63
2500	0.70	0.68	0.66	0.73	0.84	0.55	0.73	0.79	0.63	0.65	0.76	0.58
**b-value range** **(s/mm^2^)**	**MD**			**D//**			**D┴**			**FA**		
	**AUC**	**Sp**	**Se**	**AUC**	**Sp**	**Se**	**AUC**	**Sp**	**Se**	**AUC**	**Sp**	**Se**
0–1000	0.68	0.68	0.66	0.72	0.95	0.5	0.73	0.92	0.47	0.68	0.58	0.76
0–1500	0.74	0.63	0.82	0.75	0.82	0.71	0.78	0.74	0.76	0.63	0.55	0.74
0–2000	0.76	0.71	0.76	0.74	0.92	0.58	0.77	0.84	0.68	0.62	0.66	0.63
0–2500	0.73	0.68	0.79	0.72	0.84	0.58	0.73	0.55	0.82	0.67	0.84	0.47

**Table 5:** Correlation between DTI parameters and GS for each considered b-value and b-value range (adjusted *p* = 0.0014; significant correlations are highlighted in bold).

**b-Value (s/mm^2^)**	**MD**		**D//**		**D┴**		**FA**	
	**𝜌**	**P**	**𝜌**	**P**	**𝜌**	**P**	**𝜌**	**P**
500	−0.22	0.06	−0.33	0.003	−0.31	0.006	−0.18	0.13
1000	−0.29	0.01	−0.35	0.002	−0.36	0.002	−0.25	0.03
1500	−0.33	0.003	−0.37	**0.001**	−0.39	**0.0004**	−0.18	0.1
2000	−0.43	**0.0001**	−0.45	**<0.0001**	−0.49	**<0.0001**	−0.29	0.01
2500	−0.39	**0.0006**	−0.40	**0.0003**	−0.43	**0.0001**	−0.23	0.04
**b-value range** **(s/mm^2^)**	**MD**		**D//**		**D┴**		**FA**	
	**𝜌**	**P**	**𝜌**	**P**	**𝜌**	**P**	**𝜌**	**P**
0–1000	−0.33	0.004	−0.38	**0.0007**	−0.40	**0.0003**	−0.27	0.02
0–1500	−0.42	**0.0002**	−0.44	**<0.0001**	−0.49	**<0.0001**	−0.21	0.06
0–2000	−0.46	**<0.0001**	−0.44	**<0.0001**	−0.49	**<0.0001**	−0.20	0.09
0–2500	−0.41	**0.0002**	−0.40	**0.0003**	−0.42	**0.0002**	−0.28	0.02

## Data Availability

The data presented in this study are available on request from the corresponding author. MRI images and biopsies are not publicly available due to ethical restrictions and patients’ privacy. The MRI images can be made available after anonymization upon request. The code and scripts are not published in a public repository and can be made available upon request.

## Supplementary Materials

The following supporting information can be downloaded at: https://www.mdpi.com/article/10.3390/jpm13050860/s1, Figure S1: Contrast ratio (CR) of MD and FA between benign and cancerous tissue in the patients’ cohort; Table S1: Area under the curve (AUC), specificity (Sp) and sensibility (Se) obtained from ROC curve analysis for the classification of benign and PCa tissue; Figure S2: Variability of MD estimate in PCa lesions; Figure S3: ROC curves; Table S2: Pearson’s correlation (r) between DTI parameters and GS for each considered b-value and b-value range (adjusted *p* = 0.0014; significant correlations are highlighted in bold).

## Appendix A. Diffusion Model

The algorithm adopted in the extraction of the diffusion tensor (DT) models the signal attenuation as a mono-exponential decay:

S_i_(b) = S_0_ exp(−bD_i_), (A1)

where S_i_(b) is the attenuated signal intensity in the i^th^-direction, S_0_ is the image with no diffusion weighting, b is the b-value, defined as b = γ^2^g^2^δ^2^(∆−δ/3), with g as the diffusion gradient strength, δ as the diffusion gradient duration, and ∆ as the time during which the diffusion process is observed [15]. Inverting the relation in (1) in the case of a single b-value, or fitting the expression ln((S_i_(b))/S_0_) = −bD_i_ in the case of multiple b-values, the algorithm computes the one independent apparent diffusion coefficient (ADC) for each diffusion direction (D_1_, D_2_, …, D_6_, in our case). Then, by solving a system of linear equations through linear regression, and through diagonalization of the DT, it is possible to derive its eigenvalues (λ_1_, λ_2_, λ_3_) and eigenvectors (v1, v2, v3), and the parametric maps of rotationally invariant parameters, i.e., MD, FA, axial (D//), and radial (D┴) diffusivity, according to the following relations:

MD = (λ_1_ + λ_2_ + λ_3_)/3, (A2)

D// =λ_1_, (A3)

D┴ = (λ_2_ + λ_3_)/2, (A4)

(A5) $$\mathrm{FA}=\sqrt{\frac{3\left[{\left(\lambda_{1}-\mathrm{MD}\right)}^{2}+{\left(\lambda_{2}-\mathrm{MD}\right)}^{2}+{\left(\lambda_{3}-\mathrm{MD}\right)}^{2}\right]}{2({\lambda_{1}}^{2}+{\lambda_{2}}^{2}+{\lambda_{3}}^{2})}}.$$

By using the computed MD it is possible to obtain an estimate of the diffusion length l_D_ travelled on average by water molecules, also named the root mean squared displacement (RMSD):

(A6) $$l_{D}=\mathrm{RMSD}\;\sim \;\sqrt{2N\cdot \mathrm{MD}\cdot ∆}$$

with N = 3. The RMSD quantifies the intrinsic resolution of DWI investigation. The diffusion length depends on the type of the selected diffusion dynamics; the higher the b-value, the slower the dynamics of the water molecules being probed, the shorter the estimated l_D_. Regarding the experimental set-up employed in this study, considering ∆ = 50 ms, the diffusion length probed varies within a minimum of l_D_ ~ 10 µm (obtained with an average MD = 0.0006 s/mm^2^, calculated from DWIs acquired at b = 2500 s/mm^2^) and a maximum of l_D_ ~ 25 µm (obtained using b = 500 s/mm^2^, with an average MD = 0.0028 s/mm^2^).
