# Supplementary material for: Acquisition Parameters Influence Diffusion Metrics Effectiveness in Probing Prostate Tumor and Age-Related Microstructure
Source: J Pers Med. 2023 May 20;13(5):860. doi: 10.3390/jpm13050860 (PMC10222109; doi:10.3390/jpm13050860)
Supplement: Supplementary file 1 [file jpm-13-00860-s001.zip › jpm-2365879-supplementary.pdf]

Supplementary Information

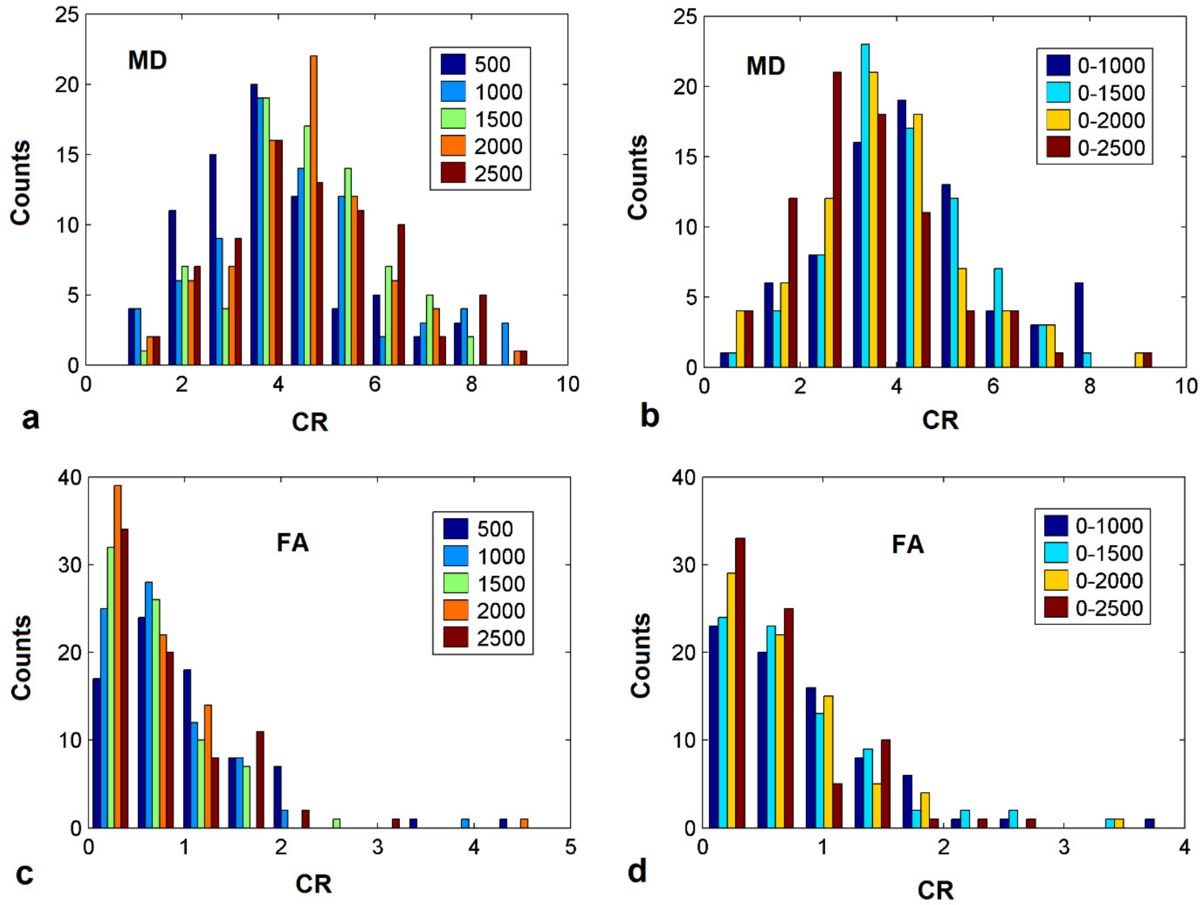

Figure S1. Contrast ratio (CR) of MD and FA between benign and cancerous tissue in the patients' cohort. Histograms of CR computed in MD and FA maps at different b-values (**a**,**c**) and b-value ranges (**b**,**d**). Most of the lesions are better visualized in MD maps obtained at b=2000 s/mm^2 while FA maps offer poor contrast, regardless of the b-value, as indicated by histograms skewed to the left.

Table S1. The area under the curve (AUC), specificity (Sp), and sensibility (Se) obtained from ROC curve analysis for the classification of benign and PCa tissue.

b-value (s/mm²)	MD			D//			D⊥			FA		
	AUC	Sp	Se	AUC	Sp	Se	AUC	Sp	Se	AUC	Sp	Se
500	1	1	0.97	1	0.97	0.99	1	0.97	1	0.84	0.7	0.86
1000	1	0.99	1	0.99	1	0.97	0.99	1	0.99	0.75	0.84	0.55
1500	1	1	1	1	1	1	1	1	1	0.7	0.79	0.57
2000	1	0.97	1	1	0.97	1	1	0.97	1	0.67	0.86	0.5
2500	1	0.99	0.99	1	0.96	1	1	0.96	1	0.8	0.87	0.62
**b-value range (s/mm²)**	**MD**			**D//**			**D⊥**			**FA**		
	**AUC**	**Sp**	**Se**	**AUC**	**Sp**	**Se**	**AUC**	**Sp**	**Se**	**AUC**	**Sp**	**Se**
0-1000	1	1	0.97	0.98	0.99	0.99	0.98	0.99	0.99	0.75	0.64	0.75
0-1500	1	0.99	1	1	0.99	1	1	0.99	1	0.64	0.33	0.89
0-2000	0.99	0.96	1	0.99	0.97	1	0.99	0.97	1	0.58	0.93	0.25
0-2500	0.98	0.91	1	0.98	0.97	0.95	0.98	0.96	0.96	0.6	0.87	0.36

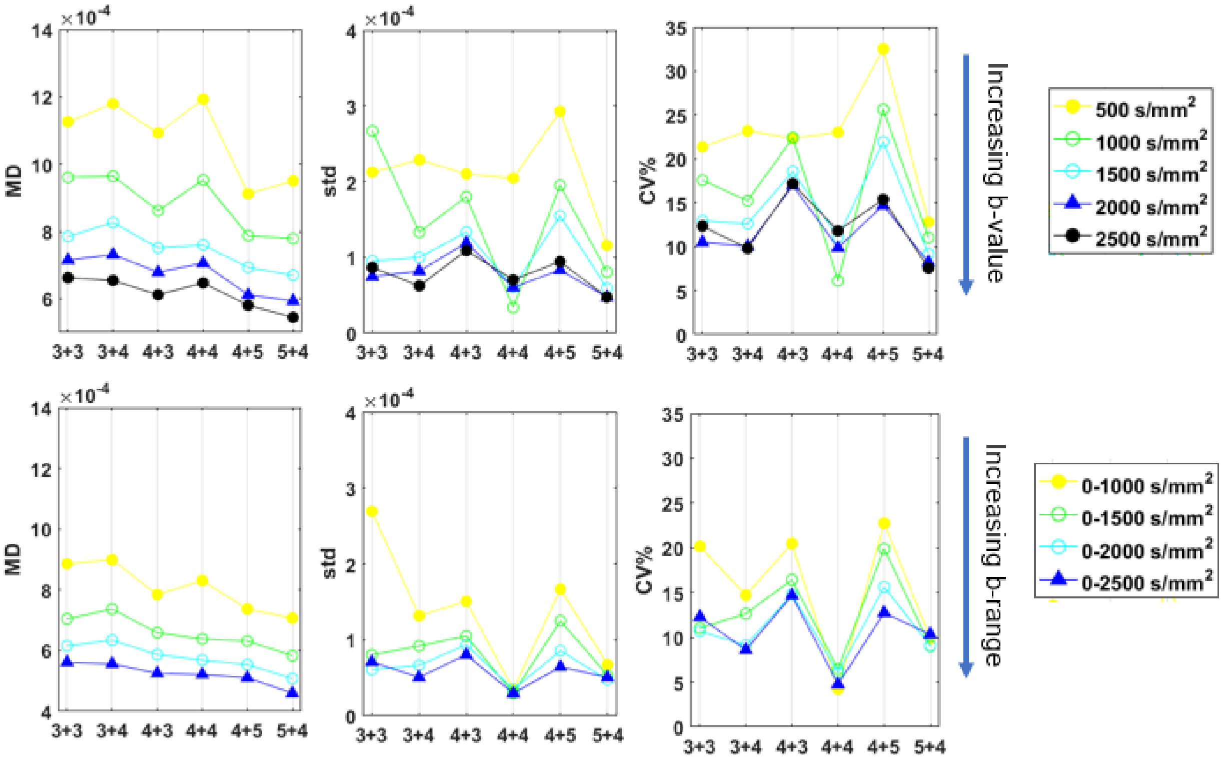

Figure S2. Variability of MD estimate in PCa lesions. MD was averaged over groups of lesions sharing the same Gleason Score, GS (**a**,**d**). Mean MD values and their standard deviations (**b**,**e**) are plotted towards b-values and b-ranges. The last column (**c**,**f**) shows the coefficient of variation, CV%. The legend indicates different b-values and b-value ranges.

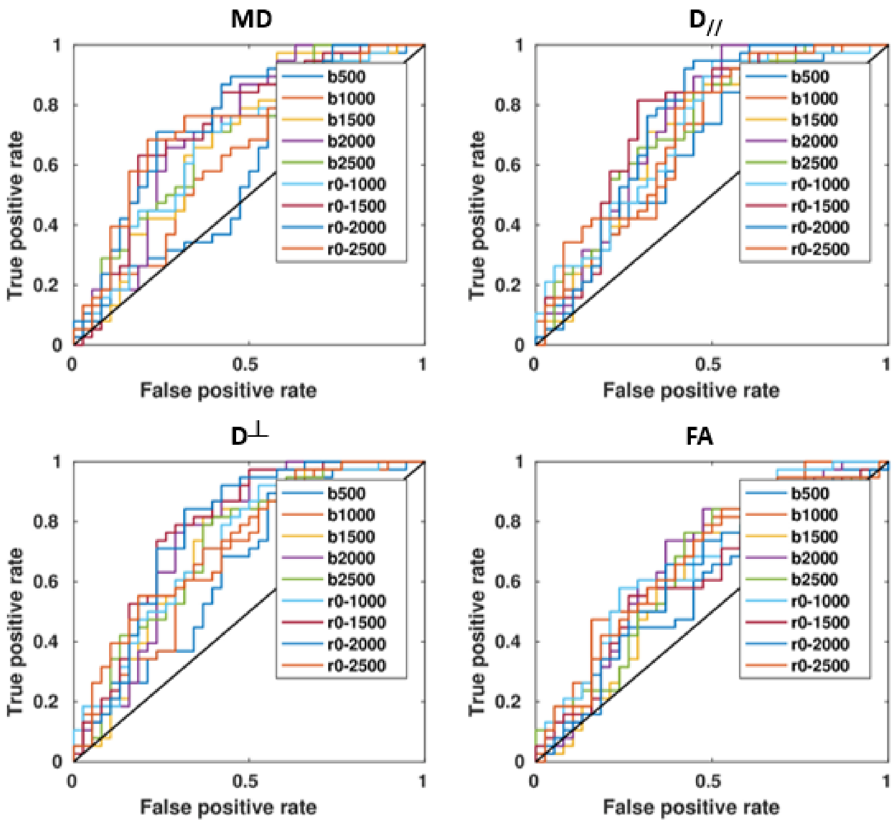

Figure S3. ROC curves. Diagnostic ability of DTI metrics (MD=mean diffusivity, D//=axial diffusivity, D⊥=radial diffusivity, FA=fractional anisotropy) in the discrimination between PCa lesions with low- vs high-Gleason grade, for each b-value (ranging from 500 to 2500 s/mm²) and b-value range (0-1000 to 0-2500 s/mm²).

Table S2. Pearson's correlation (r) between DTI parameters and GS for each considered b-value and b-value range (adjusted P = 0.0014; significant correlations are highlighted in bold).

**b-value (s/mm²)**	**MD**		**D//**		**D⊥**		**FA**	
	**r**	**P**	**r**	**P**	**r**	**P**	**r**	**P**
500	-0.25	0.03	-0.33	0.003	-0.32	0.004	-0.13	0.3
1000	-0.30	0.008	-0.35	0.005	-0.33	0.003	-0.19	0.1
1500	-0.34	0.003	-0.35	0.002	-0.39	**0.0006**	-0.12	0.3
2000	-0.43	**0.0001**	-0.45	**< 0.0001**	-0.48	**< 0.0001**	-0.21	0.07
2500	-0.39	**0.0006**	-0.42	**0.0002**	-0.44	**<0.0001**	-0.25	0.03
**b-value range (s/mm²)**	**MD**		**D//**		**D⊥**		**FA**	
	**r**	**P**	**r**	**P**	**r**	**P**	**r**	**P**
0-1000	-0.32	0.004	-0.33	0.004	-0.34	0.003	-0.24	0.04
0-1500	-0.38	**0.0007**	-0.39	**0.0005**	-0.45	**<0 .0001**	-0.19	0.1
0-2000	-0.42	**<0.0001**	-0.43	**< 0.0001**	-0.48	**<0 .0001**	-0.18	0.1
0-2500	-0.40	**0.0003**	-0.44	**<0.0001**	-0.46	**<0.0001**	-0.27	0.02
